# Metals in *Pleurozium schreberi* and *Polytrichum commune* from areas with various levels of pollution

**DOI:** 10.1007/s11356-016-6278-0

**Published:** 2016-02-24

**Authors:** Krzysztof Zawadzki, Aleksandra Samecka-Cymerman, Krzysztof Kolon, Bronisław Wojtuń, Lucyna Mróz, Alexander J. Kempers

**Affiliations:** Department of Ecology, Biogeochemistry and Environmental Protection, University of Wrocław, ul. Kanonia 6/8, 50-328 Wrocław, Poland; Institute for Water and Wetland Research, Department of Environmental Science, Radboud University Nijmegen, Huygens building, Heyendaalseweg 135, 6525 AJ Nijmegen, The Netherlands

**Keywords:** Terrestrial mosses, Endohydric, Ectohydric, Trace element

## Abstract

Metals deposited into ecosystems are non-degradable and become one of the major toxic agents which accumulate in habitats. Thus, their concentration requires precise monitoring. To evaluate pollution around a chlor-alkali plant, a glass smelter, two power plants and a ceramic and porcelain factory, we selected terrestrial mosses with different life forms: the orthotropic and endohydric *Polytrichum commune* and plagiotropic and ectohydric *Pleurozium schreberi*. Metal concentrations were determined in both species growing together at sites situated at various distances approximately 0.75, 1.5, 3 and 6 km from polluters. MARS analysis evaluated different tendencies of both species for Cd, Co and Pb accumulation depending on the distance from the emitter. In *P. schreberi*, the concentration of these metals diminished relatively rapidly with an increasing distance from the emitter up to 3000 m and then stabilised. For *P. commune*, a steady decrease could be observed with increasing the distance up to 6000 m. PCCA ordination explained that both species from the vicinity of the chlor-alkali plant were correlated with the highest Co, Cr, Cu, Fe and Pb as well as Mn and Ni concentrations in their tissues. The mosses from sites closest to both power plants were correlated with the highest Cd and Zn concentrations. *P. commune* contained significantly higher Cd, Cr, Ni, Pb and Zn concentrations compared to *P. schreberi*. This may be caused by the lamellae found in the leaves of *P. commune* which increase the surface area of the possible aerial absorption of contaminants. Soil may also be an additional source of metals, and it affects the uptake in endohydric *P. commune* more than in ectohydric *P. schreberi*. However, the precise explanation of these relations needs further investigation.

**Electronic supplementary material:**

The online version of this article (doi:10.1007/s11356-016-6278-0) contains supplementary material, which is available to authorized users.

## Introduction

Metals introduced into ecosystems usually have the potential of disturbing their chemical balance (Kabata-Pendias 2001; Pająk and Jasik 2011). Once deposited, they are non-decomposable and become incorporated in habitats (Kabata-Pendias 2001; Sardans and Peñuelas 2005). As a result of trace element emissions many soils remain polluted, which affects all food chains (Shotbolt et al. 2007). Thus, the concentration of these xenobiotics in the environment requires accurate monitoring. Among plants, bryophytes, used for the first time by Rühling and Tyler (1968), have been known as very useful accumulative bioindicators able to identify distribution trends of metals in the environment (Markert et al. 1996; Samecka-Cymerman et al. 2006; Fernández et al. 2015; Vuković et al. 2015). These plants are perennial, stationary, widespread and with convenient physiological and morphological features that enable them to accumulate considerable amounts of xenobiotics (Richardson 1981; Zechmeister et al. 2003; Ćujić et al. 2014). Additionally, their cation exchange capacity is increased by polygalacturonic acids on the external side of the cell wall and proteins in the plasma membrane (Ćujić et al. 2014).

For this investigation, two moss species with different native life-forms were selected: the vertically growing, orthotropic, endohydric *P. commune* Hedw. and horizontally growing, plagiotropic, ectohydric *Pleurozium schreberi* (Willd. ex Brid.) Mitt. *P. commune* has an internal conducting system while *P. schreberi* lacks such a system, and water is taken from the plant surface (Markert and Weckert 1993; Victoria et al. 2009). The aim of this paper is to investigate metal concentrations in *P. commune* and *P. schreberi* used as bioaccumulators in the vicinity of five emitters of various contaminant loads to compare their metal bioaccumulation abilities. Both species were employed previously in the evaluation of contamination elsewhere in Europe (Markert and Weckert 1993; Niemelä et al. 2007; Kosior et al. 2010; Šoltés and Gregušková 2013).

We tested the hypothesis that the endohydric *P. commune* contains more metals than the ectohydric *P. schreberi* in polluted areas because of the specific leaf morphology and ability to accumulate metals from soil. This report continues our previous work on the comparison of mercury accumulation in ecto- and endohydric moss species (Zawadzki et al. 2014).

## Materials and methods

### Sampling design

**Fig. 1:**
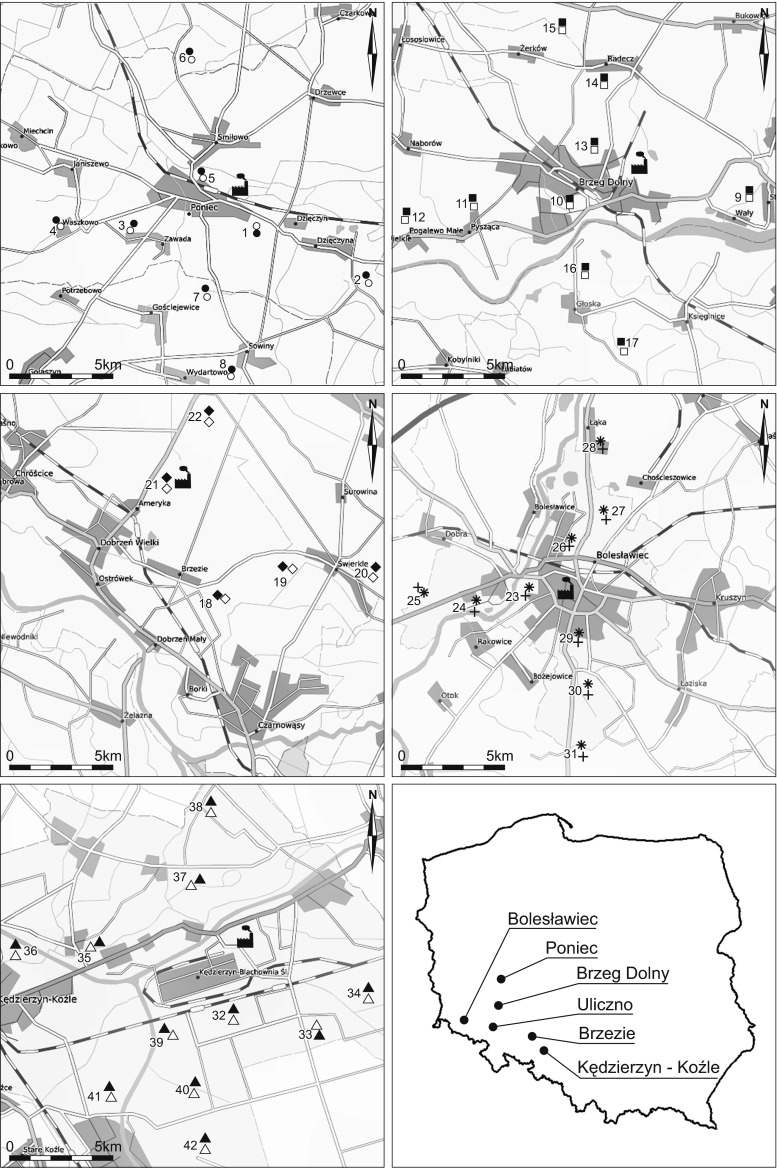
Location of the sampling sites; *empty circle* = sites of *P. schreberi, filled circle* = sites of *P. commune* from the vicinity of the glass smelter in Poniec; *empty square* = sites of *P. schreberi, filled square* = sites of *P. commune* from the vicinity of chlor-alkali industry in Brzeg Dolny; *open diamond =* sites of *P. schreberi*, *filled diamond* = sites of *P. commune* from the vicinity of the power plant in Brzezie; *cross* = sites of *P. schreberi*, *star* = sites of *P. commune* from the vicinity of the ceramics and porcelain factory in Bolesławiec; *empty triangle* = sites of *P. schreberi, filled triangle* = sites of *P. commune* from the vicinity of the power plant in Kędzierzyn-Koźle; Uliczno-control site

Five industrial polluters were selected in Poland as described by Zawadzki et al. (2014). In all the areas, the sites were only chosen where *P. schreberi* and *P. commune* grew together (Fig. 1, ESM 1 and 2). Moss samples were taken from the centre of the industrial sites along four different wind-oriented transects (N, E, S and W). The sampling sites were selected, where possible, at distances starting from as close as possible to the source of pollution (at ∼0.75 km), and then further away at 1.5, 3 and 6 km in each of the four wind directions (Fernández et al. 2000; González-Miqueo et al. 2010). However, not in all of the sampling sites did both species occur together and were thus not represented in the results of this investigation. The location of the sampling sites is given by the distance in kilometres from the centre of the polluter along the four wind directions (e.g. 1.5E means 1.5 km from the polluter eastward): (1) Glass smelter in Poniec (51° 45′ 37″ N,16° 48′ 57″ E), sampling sites 1–8 (1.5E, 6E, 1.5W, 3W, 0.75N, 3N, 3S, 6S, respectively); (2) Chlor-alkali factory in Brzeg Dolny (51° 16′ 29″ N, 16° 44′ 21″ E), sites 9–17 (6E, 0.75W, 3W, 6W, 0.75N, 1.5N, 3N,0.75S, 3S, respectively); (3) Power plant in Brzezie (50° 45′ 07″ N, 17° 53′ 16″ E), sites 18–22 (1.5E, 3E, 6E, 3W, 6W, respectively); (4) Porcelain and ceramics factory in Bolesławiec (51° 16′ 11″ N,15° 34′ 03″ E), sites 23–31 (1.5E, 3E, 6E, 0.75W, 1.5W, 3W, 0.75N, 1.3N, 6N respectively); and (5) Power plant in Kędzierzyn-Koźle (50° 21′ 29″ N, 18° 17′ 16″ E), sites 32–42 (0.75E, 3E, 6E, 3W, 6W, 3N, 6N, 0.75S, 1.5S, 3S, 6S, respectively). Unpolluted control site 43 was selected in a forest clear-cut north of the village of Uliczno (Lower Silesia N 50° 48′ 59″; E 16° 42′ 42″). At each site, we selected squares of 25 m × 25 m (Varela et al. 2010) and collected mosses randomly according to the procedures as described in Zawadzki et al. (2014). The collected number of moss samples was *N* = 43 sites × 5 replicates = 215.

### Chemical analysis

The air-dried mosses were homogenised to a fine powder in an IKA Labortechnik M20 laboratory mill and further treated as described by Kosior et al. (2015) using FAAS (Avanta PM from GBC) for Fe, Mn and Zn determination and GFAAS (PinAAcle 900Z from Perkin-Elmer) for Cd, Co, Cr, Cu, Ni and Pb determination. All the elements were compared in three replicates against standards (Sigma Chemicals Co.) and the results calculated on a dry weight basis. A full description of the method is given in Kosior et al. (2015) and Electronic Supplementary Material ESM 3.

### Statistical analysis

Shapiro-Wilk’s *W* test was used to verify the normality of the analysed features and the Brown-Forsythe test to check the homogeneity of variances (Argaç 2004). Box-Cox transformation was applied to obtain normal distribution and then ANOVA to evaluate differences between mean metal concentrations in the mosses from different sites (Zar 1999).

The significance of differences in the concentration of metals in *P. schreberi* and *P. commune* was compared with the *t* test.

A post hoc LSD test was applied to compare metal concentrations in mosses between the directions for sites situated in the vicinity of the five examined emitters.

The dependence of the element content in mosses (non-transformed data) on the distance from polluters was checked by calculating *R* coefficients with the nonparametric Spearman rank correlation (Dowdy et al. 2004). If the correlation coefficients were statistically significant (statistical significance, *P* value <0.05), nonparametric regression equations were calculated using multivariate adaptive regression splines (MARS). MARS is an implementation of techniques popularised by Friedman (1991) for solving regression‐type problems. MARS is a nonparametric regression procedure which makes no assumption about the underlying functional relationship between dependent and independent variables (Hastie et al. 2009). MARS combines classical linear regression, mathematical construction of splines and binary recursive partitioning to linear (Muñoz and Felicísimo 2004). Generalised cross validation (GCV) is a measure of the optimum choice of the model. The best model is the one with a minimum GCV value (Nash and Bradford 2001; Izenman 2008). MARS equations resulting from the relationships are shown in graphs.

Similarity of the concentration of nine metals (Cd, Co, Cr, Cu, Fe, Mn, Ni, Pb and Zn) in moss samples from 43 sites was analysed by principal component and classification analysis (PCCA) (Legendre and Legendre 1998). Mn and Ni were excluded as having the lowest correlation coefficient with first and second factors and were added as supplementary variables (Zuur et al. 2007).

All calculations were carried out using Statistica 12 software (StatSoft, Inc 2014).

## Results

The metal concentrations in *P. schreberi* and *P. commune* are shown in Tables 1 and 2, ESM 4–8. The moss samples from different sites differed significantly in the concentrations of metals (ANOVA, *p* < 0.05). The maximum concentrations of metals in *P. schreberi* from the control site (Table 2) were lower than those in *P. schreberi* from sites in the relatively unpolluted area (Kosior et al. 2010) by up to Cd 50, Cr 50, Cu 60, Fe 63, Mn 46, Ni 80, Pb 74 and Zn 19 (percentage values). The maximum concentration of metals in *P. commune* from the control site was 70 % lower than for Cu and similar for Pb compared to those in *Polytrichum strictum* examined by de Ferro et al. (2013) in the same type of clean sites. Our results thus indicate that the control site can be considered relatively free from industrial influence. The element concentrations in the examined *P. schreberi* and *P. commune* from the vicinity of five sources of pollution were significantly higher (*t* test, *P* < 0.05) than in both species from the control site. Furthermore, the upper values of metals in these mosses from the contaminated sites were higher by up to Cr 55, Cu 59, Mn 583, Pb 19 and Zn 77 (percentage values) than those measured in epigeic mosses (*Brachythecium* sp. and *Kindbergia praelonga*) investigated by Ćujić et al. (2014) near a power plant, thus indicating the presence of pollution in the examined area. *P. schreberi* and *P. commune* collected around the chlor-alkali plant contained the highest Co, Cu, Fe and Pb concentrations (post hoc LSD, *P* < 0.05) and the highest Ni concentration (post hoc LSD, *P* < 0.05) around the glass smelter in comparison with mosses for all other polluters. Similar enrichment in metal concentrations in *Brachythecium rutabulum* was reported by Kolon et al. (2015) around the same chlor-alkali plant. According to Varun et al. (2012), Ni is a colouring agent showing high accumulation in plants near glass industry sites.

**Table 1:** Minimum, maximum, median values (mg · kg^−1^) and average deviations (AD) in *P. schreberi* and *P. commune* from all the sampling sites. ANOVA calculated after Box-Cox transformation

Metal	Minimum	Maximum	Median	AD	Analysis of variance	
					F	*P*
*P. schreberi*						
Cd	0.1	1.2	0.3	0.2	4.9	<0.001
Co	0.2	1.5	0.4	0.2	14.0	<0.001
Cr	1.5	21	3.9	2.7	8.1	<0.001
Cu	8.4	51	13	7.9	25.8	<0.001
Fe	107	5228	527	658	24.8	<0.001
Mn	136	1250	491	248	13.2	<0.001
Ni	0.7	9.7	1.7	1.1	12.8	<0.001
Pb	3.9	28	7.6	4.4	14.7	<0.001
Zn	30	130	50	14.2	19.807	<0.001
*P. commune*						
Cd	0.2	1.4	0.4	0.2	12.9	<0.001
Co	0.2	1.6	0.5	0.3	34.6	<0.001
Cr	1.7	31	5.9	5.1	11.3	<0.001
Cu	11	59	16	7.9	45.7	<0.001
Fe	290	5548	692	745	51.4	<0.001
Mn	126	1367	460	234	96.3	<0.001
Ni	1.1	9.9	2.7	1.3	19.8	<0.001
Pb	4.9	32	9.5	4.9	36.3	<0.001
Zn	37	138	59	15	24.127	<0.001

**Table 2:** Minimum, maximum, median values (mg · kg^−1^) and average deviations (AD) in *P. schreberi* and *P. commune* from the control site

Metal	Minimum	Maximum	Median	AD
*P. schreberi*				
Cd	0.2	0.3	0.2	0.03
Co	0.2	0.3	0.3	0.1
Cr	1.1	2.6	1.7	0.4
Cu	2.0	5.0	4.0	0.9
Fe	208	241	213	11
Mn	219	276	241	16
Ni	0.1	0.9	0.3	0.1
Pb	2.1	2.9	2.4	0.2
Zn	29	35	33	2.0
*P. commune*				
Cd	0.2	0.3	0.2	0.03
Co	0.2	0.4	0.2	0.03
Cr	1.0	2.8	1.7	0.5
Cu	3.0	6.0	4.0	1.0
Fe	246	298	255	17
Mn	257	309	271	16
Ni	0.2	0.3	0.2	0.03
Pb	0.2	0.3	0.2	0.03
Zn	31	35	32	1.4

## Discussion

### Comparison of the biomonitoring potential of *P. commune* and *P. schreberi*

Comparison of metal concentrations between *P. schreberi* and *P. commune* with the *t* test (*P* < 0.05) revealed that *P. commune* contained significantly higher Cd, Cr, Ni, Pb and Zn concentrations. Busuoic et al. (2012) and Kłos et al. (2014) evaluated the highest values of the sorption properties of some metals for *P. commune* in comparison with other moss species. However, Rumyantsev et al. (2014) argue that *P. schreberi* has the highest ability for the accumulation of especially Cu in comparison with *P. commune*. In this investigation, there was no difference in Cu and also Co, Fe and Mn concentrations between both species. According to Glime (2007) and Goffinet and Shaw (2009) *Polytrichum* leaves have lamellae that exert a positive effect on their surface area and may provide an additional way for the absorption of contaminants. Alternatively, endohydric *P. commune* may accumulate metals not only by aerial deposition but also from soil. This may explain not only the higher values observed in *P. commune* but also the fact that the gradient is visible at a greater distance. Additionally, if the pollution present is lower than in the past, the excessive metal levels from the previous emissions are still available in surface soil and may affect the uptake in *P. commune* more than in *P. schreberi*. However, Glime (2007) think that endohydric *P. commune* relies mostly on ectohydric transport. This species usually moves water externally along its stems (Trachtenberg and Zamski 1979). Eschrich and Steiner (1968) point to the existence of a loose contact between the axial conducting system and the leaf traces in *P. commune*. According to Glime (2007), *P. commune* may be ectohydric under moderately moist conditions but predominantly endohydric in dry air which stimulates high evaporative flux. There is also reported the existence of transport between stems of *P. commune* by underground rhizomes (Potter et al. 1995). However, Trachtenberg and Zamski (1979) note that water absorption through the rhizome in *P. commune* is less efficient than through the aerial surface of the gametophyte. The precise answer why *P. commune* accumulates more of certain metals than *P. schreberi* needs further investigation.

### Element enrichment in the moss depending on distance from polluters

A post hoc LSD test (*P* < 0.05) indicated that there was no difference between directions (N, E, S and W) in the concentration of metals (for pooled data per direction) in either of the examined species. This may probably be explained by the fact that all polluters are found in highly heteromorphic anthropogenic areas, partially sheltered by forests or buildings that exert influence on the local change of direction of prevailing westerly winds (Mochida et al. 2008; Wagner and Mathur 2013).

**Fig. 2:**
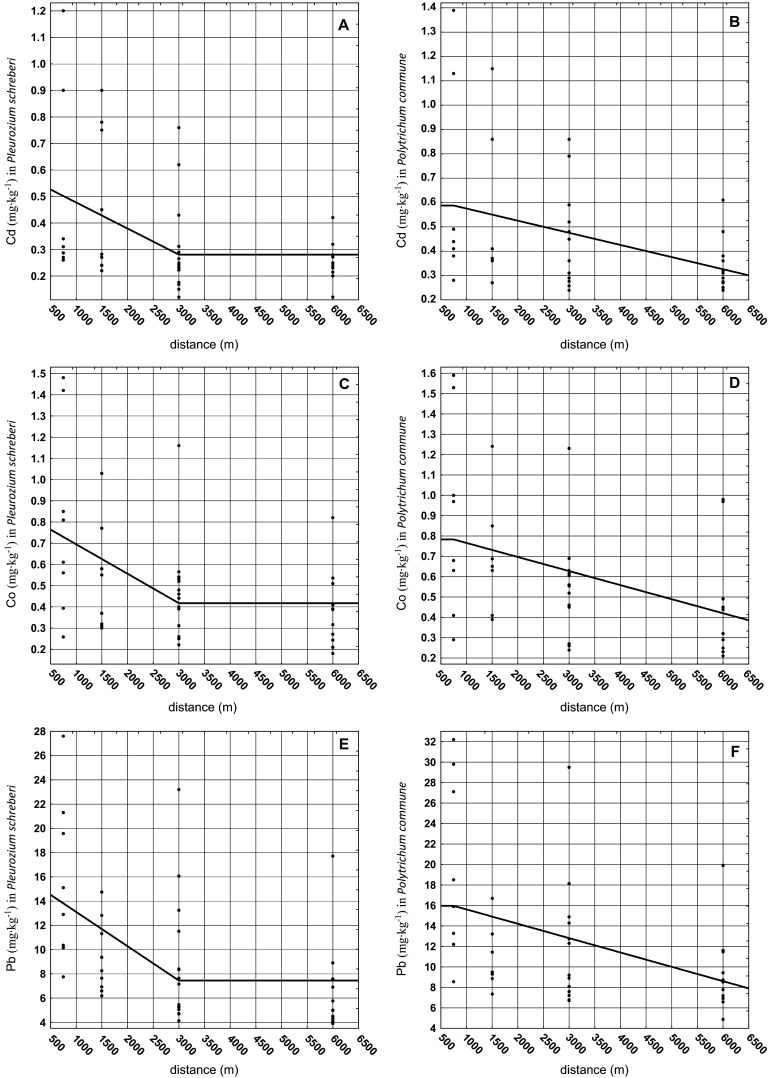
Graph with the MARS equation based on the relationship between the concentration of Cd, Co and Pb in *P. schreberi* and *P. commune* and the distance from emitters

Relations between the concentration of metals in *P. schreberi* and *P. commune* depending on the distance from the emitter (for pooled data per distance) based on nonparametric regression equations (Table 3) calculated by MARS are shown in Fig. 2. Models calculated for Cd and Co with the lowest GCV values (Table 3) reflect this trend most accurately (Nash and Bradford 2001; Izenman 2008). For Cd, Co and Pb, we found a tendency dependent on the moss species. For *P. schreberi* Cd, Co and Pb concentrations in plants diminished relatively rapidly with an increasing distance from the emitter up to 3000 m and then stabilised (Fig. 2a, c, e). For *P. commune*, a steady decrease could be observed with an increasing distance up to 6000 m (Fig. 2b, d, f). Thus, both species reveal different trends in the accumulation of metals in highly polluted environments. *P. schreberi* likely accumulated metals as long as their concentration in the environment was higher than a certain threshold value that was obviously lower at a distance higher than 3000 m. *P. commune* accumulated metals proportionally to the concentration in the environment. Mazur et al. (2013) report that *P. schreberi* contained higher Co concentrations with an increasing distance from the source of pollution, while Cd in this species did not show a clear relation with the distance. According to Shaw (1994), Goffinet and Shaw (2009), elements present at elevated levels in the environment are often found at high concentrations in bryophytes, which indicates their uptake. Some mosses can accumulate potentially toxic levels of metals (Wells and Brown 1995). Varela et al. (2015) stated that Cd and Pb exhibit significant correlations between concentrations in deposits and the moss tissue. According to these authors, the uptake or retention of metals by mosses is determined by the presence of different functional groups on the cell wall (Varela et al. 2015). Goffinet and Shaw (2009) report that sequestration onto a cation exchanger is probably an important first stage in the absorption of metals into the living protoplasts of mosses. Control of the passage of the element is provided and transport is possible when ion channels are ungated (Goffinet and Shaw 2009). Ziembik et al. (2013) suppose that the chemical properties of deposited metals affect their retention in mosses. Rao (1982) notes that terrestrial mosses may stop the uptake of a metal to which they are exposed, which allows them to survive in unfavourable conditions. It may be true that the examined *P. schreberi* develops a protective mechanism against the accumulation of elevated metal levels which settle in the close vicinity of the emitter or reduces the metal load by changing the molecular properties of enzymes on the membrane (Fernández et al. 2000). The explanation of these differences in the pattern of metal accumulation by both species needs further investigation.

**Table 3:** Results of the Spearman correlation and multivariate adaptive regression splines (MARS) for a model of relations between metal concentrations in *P. schreberi* and *P. commune* depending on the distance (750, 1500, 3000 and 6000 m) from the emitter

Element	Species	*R* Spearman correlations	Statistical significance *P* value	Equation	GCV error	Graph
Cd	*Pleurozium schreberi*	−0.373	<0.05	Cd = 2.808e−1 + 9.822e−5*max (0; 3e + 3−X)	0.052328	A
Cd	*Polytrichum commune*	−0.403	<0.05	Cd = 5.877e−1−4.987e−5*max (0; X−7.5e + 2)	0.073404	B
Co	*Pleurozium schreberi*	−0.451	<0.005	Co = 4.174e−1 + 1.387e-4*max (0; 3e + 3−X)	0.079755	C
Co	*Polytrichum commune*	−0.443	<0.005	Co = 7.830e−1−6.912e−5*max (0; X−7.5e + 2)	0.11569	D
Pb	*Pleurozium schreberi*	−0.578	<0.0005	Pb = 7.445e + 0 + 2.830e−3*max (0; 3e + 3−X)	27.07861	E
Pb	*Polytrichum commune*	−0.487	<0.005	Pb = 1.597e + 1−1.402e−3*max (0; X−7.5e + 2)	45.13515	F

**Fig. 3:**
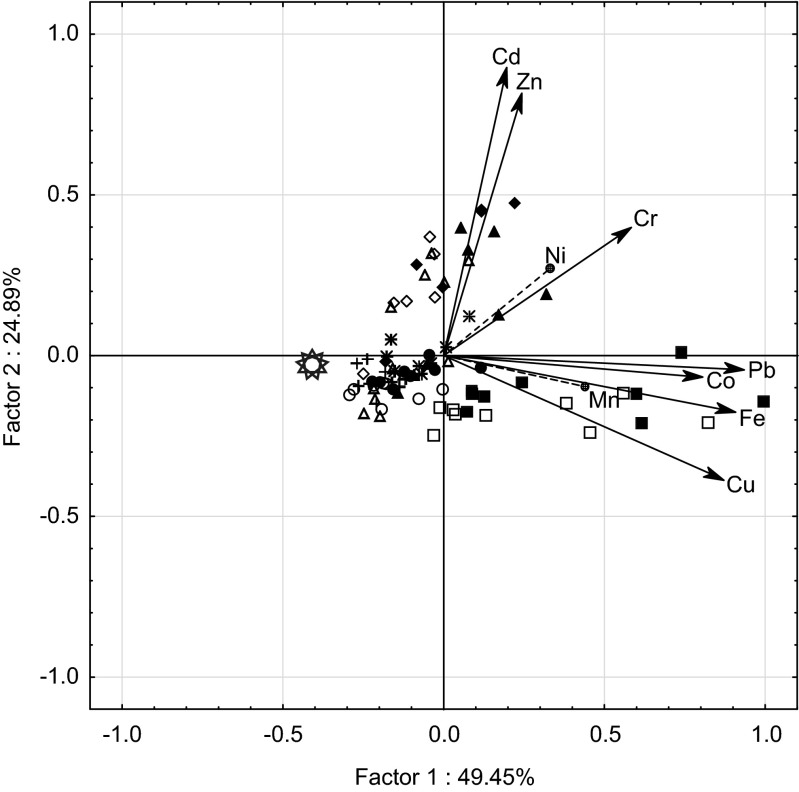
Ordination plot of *P. commune* and *P. schreberi* based on concentrations of the nine metals: Cd, Co, Cr, Cu, Fe, Pb and Zn (Mn and Ni as supplementary variables) and projection of element concentrations on the component plane; *sun* = control, other symbols refer to Fig. 1

PCCA ordination confirms our results for 43 sites of *P. schreberi* and *P. commune* (Fig. 3). The first principal component discriminates both species collected in the vicinity of the chlor-alkali factory (positive scores). The second principal component discriminates mosses at sites closest to both power plants and the ceramics and porcelain factory. The plot shows that the mosses at sites influenced by the chlor-alkali factory with positive scores of factor one contained the highest Co, Cr, Cu, Fe and Pb as well as Mn and Ni concentrations in their tissues. Mosses from the sites closest to both power plants with positive scores of factor two contained the highest Cd and Zn concentrations in their tissues. The same chlor-alkali industry was reported as a source of Co, Cr, Fe and Ni whose concentration in *B. rutabulum* increased with the decreasing distance from the factory (Kolon et al. 2015). The elevated metal levels which distinguish mosses in the vicinity of power plants are in agreement with Suchara et al. (2011) and suggest that the metals tested were significantly accumulated in part of the fly coal ash emitted by combustion plants and transported in large amounts over long distances in the air. The area of the Czech Republic influenced by the former Black Triangle emission was characterised by the highest deposition loads of metals, such as Cd and Zn (Suchara et al. 2011). Furthermore, Nagajyoti et al. (2010) report that power stations such as coal-burning power plants are sources of Cd and Zn, to name a few only, in the environment.

## Conclusions

The vicinity of the chlor-alkali plant was polluted with Co, Cr, Cu, Fe, Pb as well as Mn and Ni, which was reflected in the elevated concentration of these metals in the locally growing *P. commune* and *P. schreberi*.

The MARS model for *P. commune* and *P. schreberi* revealed that both species differed from each other in the pattern of Cd, Co and Pb accumulation in relation to the distance from the emitter.

*P. commune* contained significantly higher Cd, Cr, Ni, Pb and Zn concentrations than *P. schreberi* probably because of its surface morphology, which may enhance the capture of the metal from the atmosphere and because of the possible additional accumulation from the soil by an internal system of water transport.

The PCCA classifies the concentration of metals in *P. commune* and *P. schreberi*, which permits differentiation between chlor-alkali industry and power plants as sources of pollution.

Owing to the results presented in this report, conclusions can be made about the pollution level depending on the metal concentrations observed in *P. commune* and *P. schreberi*.

## Electronic Supplementary Material

Below is the link to the electronic supplementary material.

ESM 1 *Pleurozium schreberi* (GIF 3767 kb)

High Resolution (TIF 34106 kb)

ESM 2 *Polytrichum commune* (GIF 6534 kb)

High Resolution (TIF 60051 kb)

ESM 3 Analysis of certified reference material (PDF 161 kb)

ESM 4 Minimum, maximum, median values (mg · kg^−1^) and average deviations (AD) in *P.schreberi* and *P. commune* from Poniec sites 1–8 influenced by glass industry (PDF 755 kb)

ESM 5 Minimum, maximum, median values (mg · kg^−1^) and average deviations (AD) in *P. schreberi* and *P. commune* from Brzeg Dolny, sites 9–17 influenced by chlor-alkali industry (PDF 760 kb)

ESM 6 Minimum, maximum, median values (mg · kg^−1^) and average deviations (AD) in *P. schreberi* and *P. commune* from Brzezie sites 18–23 influenced by power plant (PDF 830 kb)

ESM 7 Minimum, maximum, median values (mg · kg^−1^) and average deviations (AD) in *P. schreberi* and *P. commune* from Bolesławiec sites 24–32 influenced by porcelain plant (PDF 1078 kb)

ESM 8 Minimum, maximum, median values (mg · kg^−1^) and average deviations (AD) in *P. schreberi* and *P. commune* from Kȩdzierzyn sites 33–43 influenced by power plant (PDF 887 kb)
