# Supplementary figures and images for: Metals in Pleurozium schreberi and Polytrichum commune from areas with various levels of pollution
Source: Environ Sci Pollut Res Int. 2016 Feb 24;23:11100–8. doi: 10.1007/s11356-016-6278-0 (PMC4884573; doi:10.1007/s11356-016-6278-0)

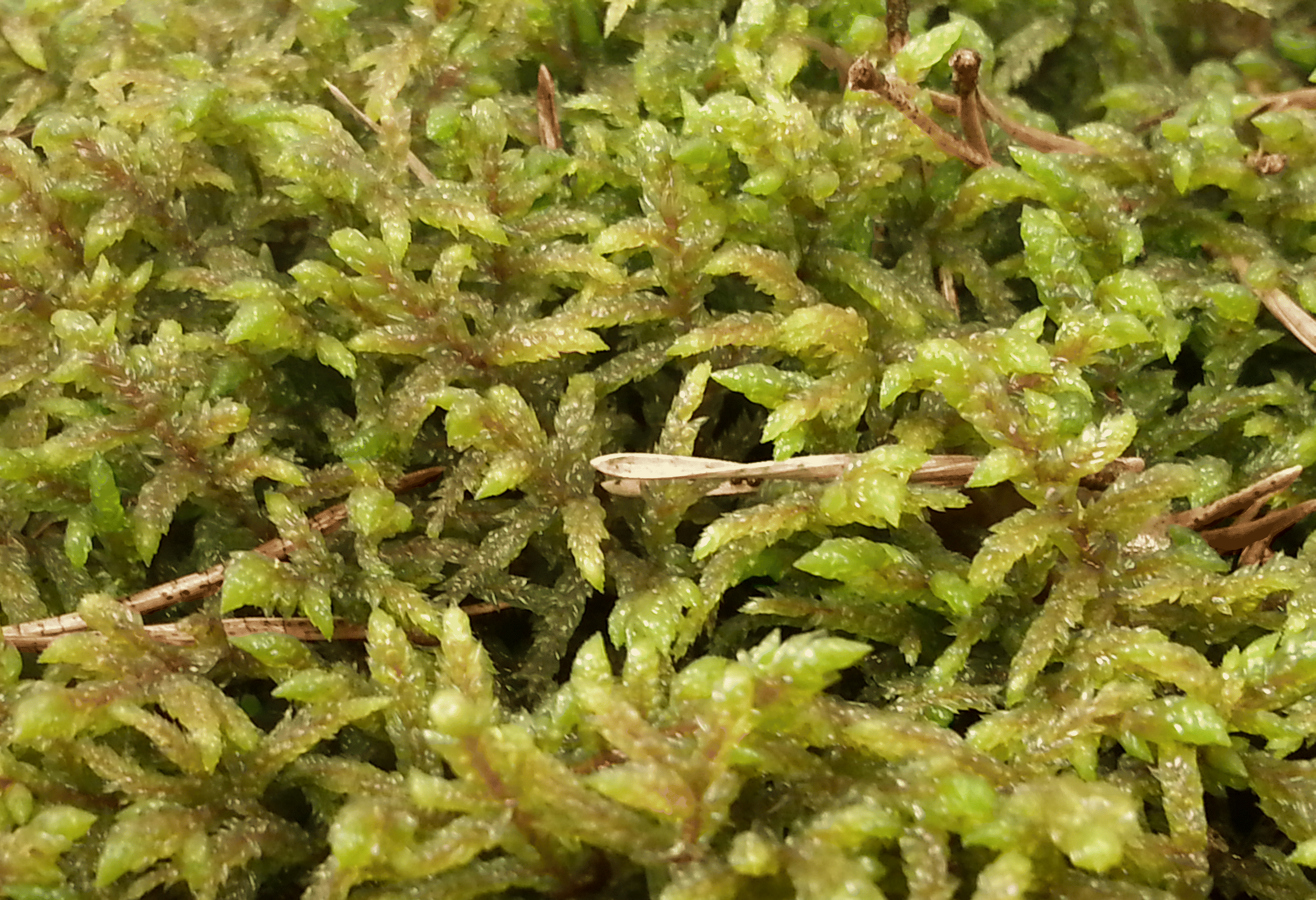

Supplement: Supplementary file 1 — Pleurozium schreberi (GIF 3767 kb) [file 11356_2016_6278_Fig4_ESM.gif]

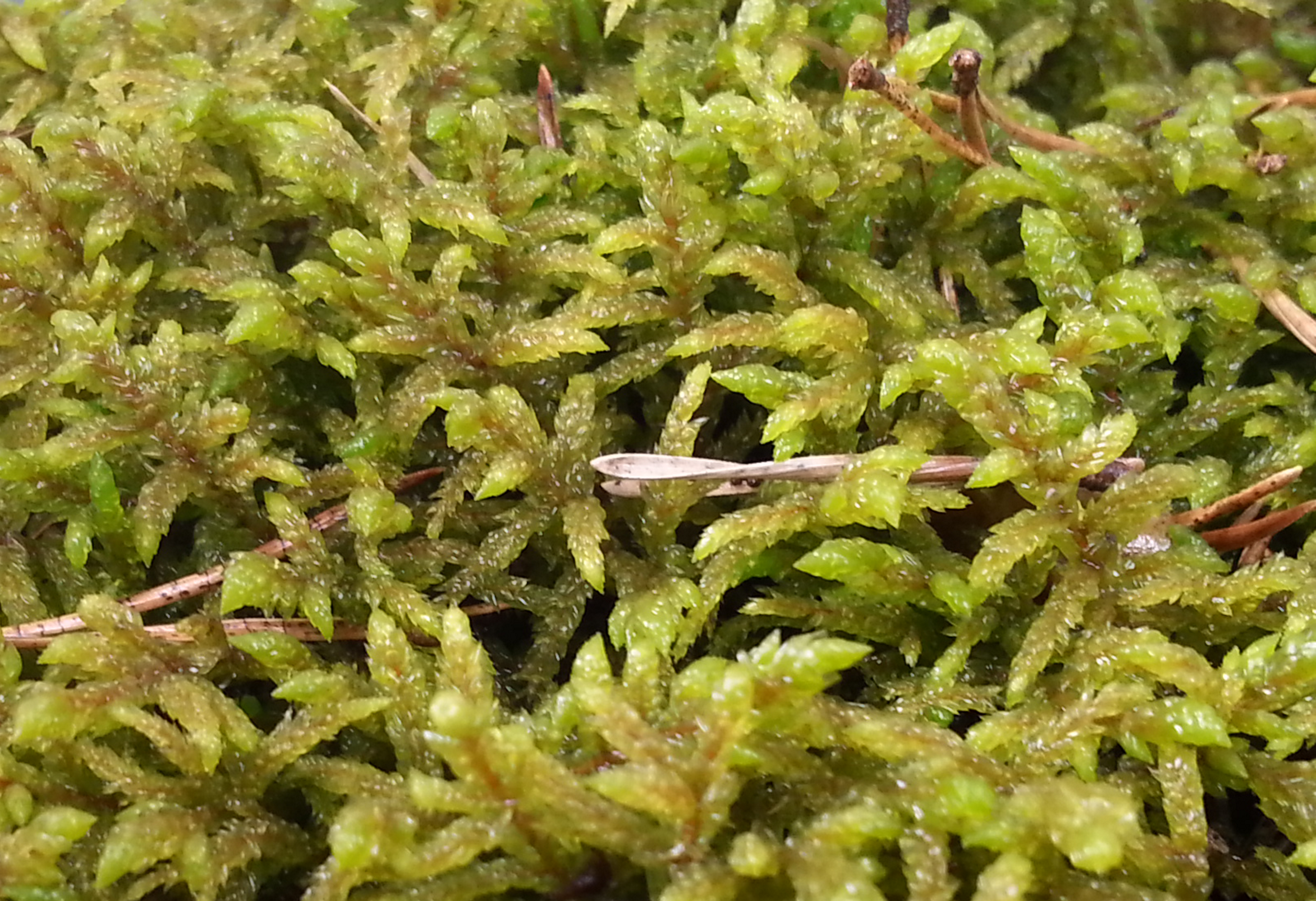

Supplement: Supplementary file 2 — High Resolution (TIF 34106 kb) [file 11356_2016_6278_MOESM1_ESM.tif]

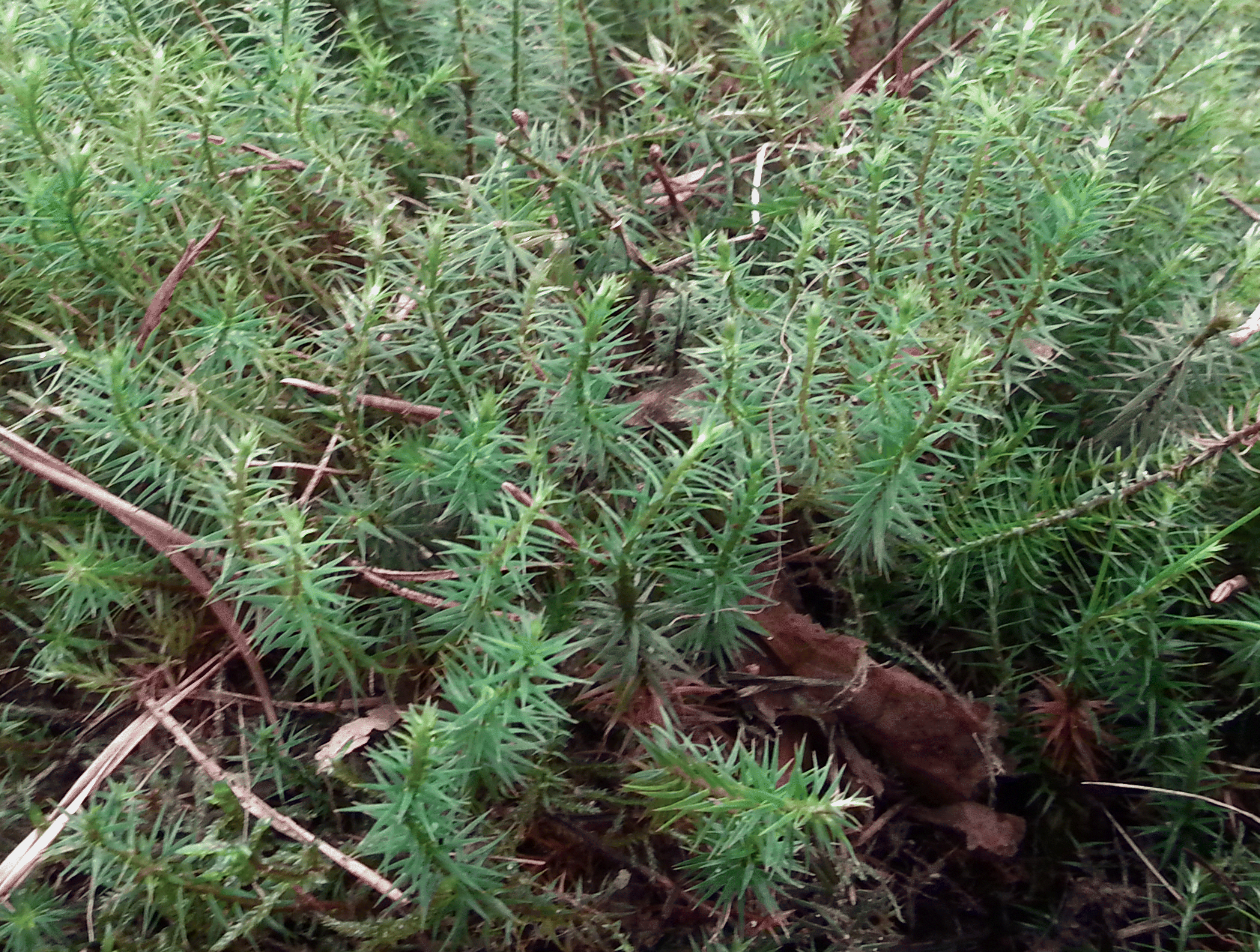

Supplement: Supplementary file 3 — Polytrichum commune (GIF 6534 kb) [file 11356_2016_6278_Fig5_ESM.gif]

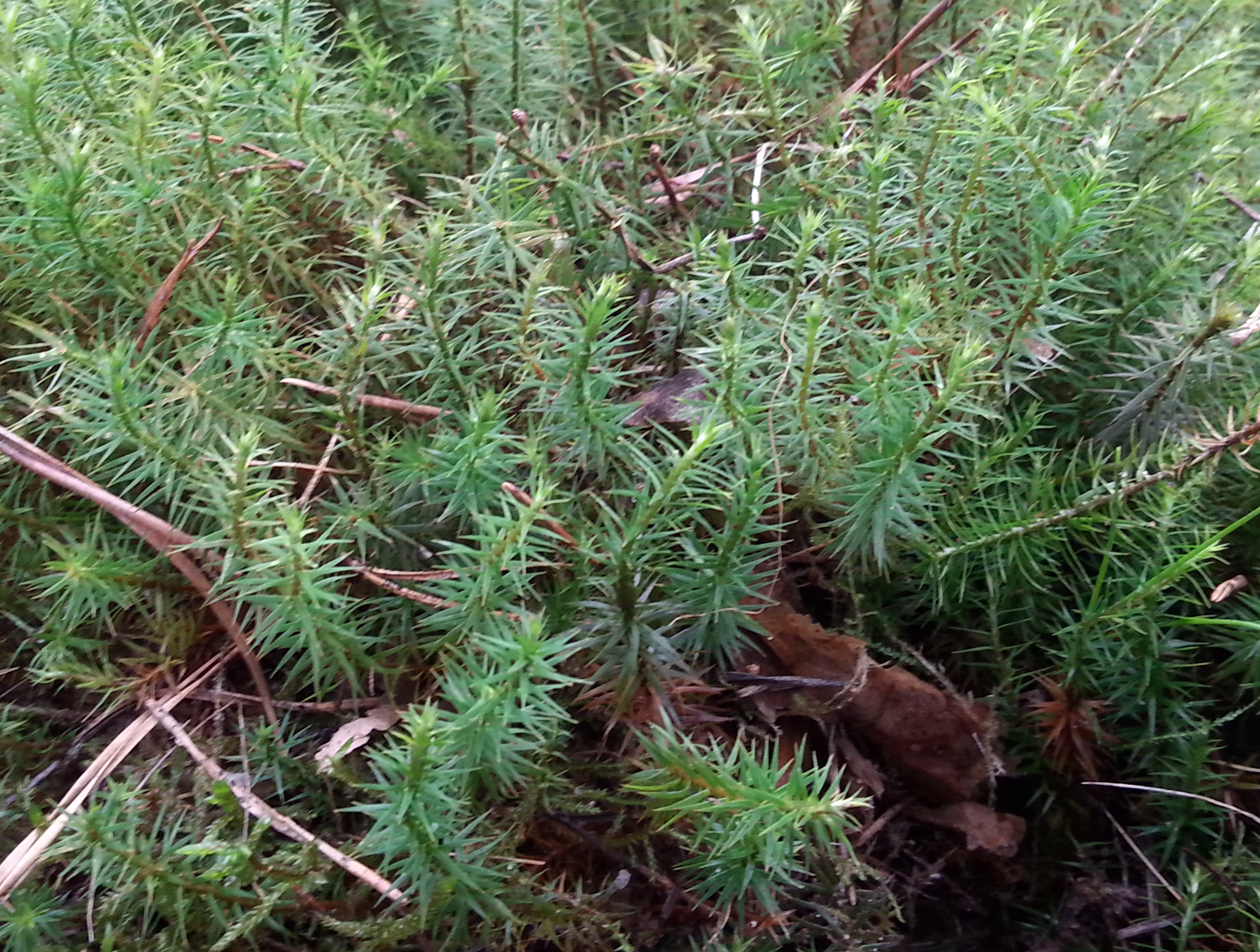

Supplement: Supplementary file 4 — High Resolution (TIF 60051 kb) [file 11356_2016_6278_MOESM2_ESM.tif]
