# Supplementary material for: Metals in Pleurozium schreberi and Polytrichum commune from areas with various levels of pollution
Source: Environ Sci Pollut Res Int. 2016 Feb 24;23:11100–8. doi: 10.1007/s11356-016-6278-0 (PMC4884573; doi:10.1007/s11356-016-6278-0)
Supplement: Supplementary file 5 — Analysis of certified reference material (PDF 161 kb) [file 11356_2016_6278_MOESM3_ESM.pdf]

ESM 3. Analysis of certified reference material

Element	Moss M2 (Finnish Forest Research Institute) standards				Moss M3 (Finnish Forest Research Institute) standards			
	Certified (mg·kg^{-1})	Found (mg·kg^{-1})	Recovery (%)	CV (%)	Certified (mg·kg^{-1})	Found (mg·kg^{-1})	Recovery (%)	CV (%)
Cd	0.454	0.459	101.10	2.6	0.106	0.108	101.89	3.7
Co	0.98	0.99	101.02	2.9	0.115	0.111	96.52	3.6
Cr	0.97	1.02	105.15	2.9	0.67	0.72	107.46	4.2
Cu	67.7	68.3	100.89	2.2	3.76	3.82	101.60	3.4
Fe	262	258	98.47	4.7	138	134	97.10	4.8
Mn	342	340	99.42	3.2	535	539	100.75	3.3
Ni	16.3	16.6	101.84	1.8	0.95	0.99	104.21	3.0
Pb	6.37	6.29	98.74	3.8	3.33	3.29	98.80	3.0
Zn	36.1	36.5	101.11	2.2	25.4	25.9	101.97	2.7
