# Supplementary material for: Metals in Pleurozium schreberi and Polytrichum commune from areas with various levels of pollution
Source: Environ Sci Pollut Res Int. 2016 Feb 24;23:11100–8. doi: 10.1007/s11356-016-6278-0 (PMC4884573; doi:10.1007/s11356-016-6278-0)
Supplement: Supplementary file 6 — Minimum, maximum, median values (mg · kg−1) and average deviations (AD) in P.schreberi and P. commune from Poniec sites 1–8 influenced by glass industry (PDF 755 kb) [file 11356_2016_6278_MOESM4_ESM.pdf]

ESM 4. Minimum, maximum, median values ($mg \cdot kg^{-1}$) and average deviations (AD) in *P. schreberi* and *P. commune* from Poniec sites 1-8 influenced by glass industry

Metal	Minimum	Maximum	Median	AD
		P. schreberi		
Cd	0.2	0.3	0.2	0.01
Co	0.2	1.0	0.3	0.1
Cr	2.8	6.5	4.3	1.3
Cu	9.5	26	13	4.9
Fe	107	520	446	95
Mn	295	942	500	156
Ni	1.0	9.7	1.7	0.9
Pb	4.3	10	5.4	1.8
Zn	35	50	42	4.2
		P. commune		
Cd	0.2	0.4	0.3	0.02
Co	0.2	1.2	0.4	0.2
Cr	3.1	10.3	5.9	1.9
Cu	13	24	17	2.9
Fe	290	668	531	103
Mn	241	785	417	167
Ni	1.1	9.9	1.9	1.3
Pb	6.6	13.3	7.4	2.0
Zn	37	67	53	6.2
