# Supplementary material for: Metals in Pleurozium schreberi and Polytrichum commune from areas with various levels of pollution
Source: Environ Sci Pollut Res Int. 2016 Feb 24;23:11100–8. doi: 10.1007/s11356-016-6278-0 (PMC4884573; doi:10.1007/s11356-016-6278-0)
Supplement: Supplementary file 7 — Minimum, maximum, median values (mg · kg−1) and average deviations (AD) in P. schreberi and P. commune from Brzeg Dolny, sites 9–17 influenced by chlor-alkali industry (PDF 760 kb) [file 11356_2016_6278_MOESM5_ESM.pdf]

ESM 5. Minimum, maximum, median values ($mg \cdot kg^{-1}$) and average deviations (AD) in *P. schreberi* and *P. commune* from Brzeg Dolny, sites 9-17 influenced by chlor-alkali industry

Metal	Minimum	Maximum	Median	AD
		P. schreberi		
Cd	0.1	0.3	0.3	0.04
Co	0.4	1.5	0.8	0.3
Cr	2.7	16	5.1	2.6
Cu	24	51	30	7.9
Fe	505	5228	1465	472
Mn	323	1250	735	288
Ni	1.1	5.6	2.0	1.2
Pb	7.5	28	1.0	4.5
Zn	30	62	48	7.7
		P. commune		
Cd	0.3	0.5	0.4	0.1
Co	0.5	1.6	0.9	0.3
Cr	2.9	20	6.2	2.6
Cu	25	59	34	8.9
Fe	686	5548	1860	522
Mn	376	1367	706	286
Ni	1.5	5.9	3.5	1.1
Pb	8.5	32	20	6.9
Zn	37	78	59	8.0
