# Supplementary material for: Metals in Pleurozium schreberi and Polytrichum commune from areas with various levels of pollution
Source: Environ Sci Pollut Res Int. 2016 Feb 24;23:11100–8. doi: 10.1007/s11356-016-6278-0 (PMC4884573; doi:10.1007/s11356-016-6278-0)
Supplement: Supplementary file 8 — Minimum, maximum, median values (mg · kg−1) and average deviations (AD) in P. schreberi and P. commune from Brzezie sites 18–23 influenced by power plant (PDF 830 kb) [file 11356_2016_6278_MOESM6_ESM.pdf]

ESM 6. Minimum, maximum, median values ($mg \cdot kg^{-1}$) and average deviations (AD) in *P. schreberi* and *P. commune* from Brzezie sites 18-22 influenced by power plant

Metal	Minimum	Maximum	Median	AD
		P. schreberi		
Cd	0.3	0.8	0.6	0.2
Co	0.2	0.5	0.3	0.1
Cr	4.4	21	8.7	4.3
Cu	8.4	10	9.0	0.6
Fe	402	878	593	154
Mn	136	334	230	58
Ni	1.4	4.5	2.0	1.1
Pb	5.8	7.6	7.6	0.6
Zn	47	108	71	18
		P. commune		
Cd	0.4	0.9	0.8	0.2
Co	0.3	0.6	0.4	0.1
Cr	5.2	31	9.5	4.6
Cu	12	15	14	0.8
Fe	489	1289	743	294
Mn	126	349	223	71
Ni	1.9	5.6	2.7	1.5
Pb	7.8	13	8.9	1.9
Zn	51	126	85	18
