# Supplementary material for: Metals in Pleurozium schreberi and Polytrichum commune from areas with various levels of pollution
Source: Environ Sci Pollut Res Int. 2016 Feb 24;23:11100–8. doi: 10.1007/s11356-016-6278-0 (PMC4884573; doi:10.1007/s11356-016-6278-0)
Supplement: Supplementary file 9 — Minimum, maximum, median values (mg · kg−1) and average deviations (AD) in P. schreberi and P. commune from Bolesławiec sites 24–32 influenced by porcelain plant (PDF 1078 kb) [file 11356_2016_6278_MOESM7_ESM.pdf]

ESM 7. Minimum, maximum, median values ($mg \cdot kg^{-1}$) and average deviations (AD) in *P. schreberi* and *P. commune* from Bolesławiec sites 23-31 influenced by porcelain plant

Metal	Minimum	Maximum	Median	AD
		P. schreberi		
Cd	0.2	0.9	0.3	0.1
Co	0.2	0.8	0.6	0.1
Cr	1.7	3.8	2.7	0.6
Cu	10	21	14	2.5
Fe	346	812	492	91
Mn	160	989	435	147
Ni	0.7	1.8	1.6	0.3
Pb	4.1	11	7.8	2.0
Zn	40	130	52	16
		P. commune		
Cd	0.3	1.2	0.4	0.2
Co	0.3	1.0	0.6	0.1
Cr	1.7	25	4.1	2.5
Cu	17	25	18	2.6
Fe	357	937	690	147
Mn	167	978	433	148
Ni	1.1	2.9	2.0	0.4
Pb	4.9	13	8.7	2.2
Zn	47	138	56	19
