# Supplementary material for: Metals in Pleurozium schreberi and Polytrichum commune from areas with various levels of pollution
Source: Environ Sci Pollut Res Int. 2016 Feb 24;23:11100–8. doi: 10.1007/s11356-016-6278-0 (PMC4884573; doi:10.1007/s11356-016-6278-0)
Supplement: Supplementary file 10 — Minimum, maximum, median values (mg · kg−1) and average deviations (AD) in P. schreberi and P. commune from Kȩdzierzyn sites 33–43 influenced by power plant (PDF 887 kb) [file 11356_2016_6278_MOESM8_ESM.pdf]

ESM 8. Minimum, maximum, median values ($mg \cdot kg^{-1}$) and average deviations (AD) in *P. schreberi* and *P. commune* from Kędzierzyn sites 32-42 influenced by power plant

Metal	Minimum	Maximum	Median	AD
		P. schreberi		
Cd	0.1	1.2	0.3	0.2
Co	0.3	1.2	0.4	0.1
Cr	1.5	7.1	2.8	1.7
Cu	9.9	17	11	2.0
Fe	425	929	625	109
Mn	422	965	503	220
Ni	1.0	4.2	1.9	0.8
Pb	3.9	15	6.9	3.2
Zn	35	91	52	16
		P. commune		
Cd	0.2	1.4	0.5	0.2
Co	0.2	1.2	0.5	0.1
Cr	2.8	25	5.4	2.4
Cu	11	19	14	2.0
Fe	509	1767	749	295
Mn	396	979	539	209
Ni	1.2	5.1	2.8	1.0
Pb	6.9	19	9.4	3.1
Zn	41	98	60	14
